# Group B streptococcus colonization in pregnancy and neonatal outcomes: a three-year monocentric retrospective study during and after the COVID-19 pandemic

**DOI:** 10.1186/s13052-024-01738-2

**Published:** 2024-09-13

**Authors:** Gregorio Serra, Lucia Lo Scalzo, Maria Giordano, Mario Giuffrè, Pietro Trupiano, Renato Venezia, Giovanni Corsello

**Affiliations:** Department of Health Promotion, Mother and Child Care, Internal Medicine and Medical Specialties “Giuseppe D’Alessandro”, University of Palermo, Palermo, Italy

**Keywords:** *Streptococcus agalactiae*, Vaginal rectal swabs, GBS colonization, *Intrapartum* antibiotic prophylaxis, Neonatal sepsis, COVID-19

## Abstract

**Background:**

Group B *Streptococcus* (GBS) is a major cause of sepsis and meningitis in newborns. The Centers for Disease Control and Prevention (CDC) recommends to pregnant women, between 35 and 37 weeks of gestation, universal vaginal-rectal screening for GBS colonization, aimed at *intrapartum* antibiotic prophylaxis (IAP). The latter is the only currently available and highly effective method against early onset GBS neonatal infections. Since the onset of the coronavirus disease 2019 (COVID-19) pandemic, the preventive measures implemented to mitigate the effects of SARS-CoV-2 infection led to the reduction in the access to many health facilities and services, including the obstetric and perinatal ones. The purpose of the present study was to evaluate the prevalence of maternal GBS colonization, as well as use of IAP and incidence of episodes of neonatal GBS infection when antibiotic prophylaxis has not been carried out in colonized and/or at risk subjects, in a population of pregnant women during (years 2020–2021) and after (year 2022) the COVID-19 pandemic, also with the aim to establish possible epidemiological and clinical differences in the two subjects’ groups.

**Methods:**

We retrospectively analyzed the clinical data of pregnant women admitted to, and delivering, at the Gynaecology and Obstetrics Unit, Department of Sciences for Health Promotion and Mother and Child Care, of the University Hospital of Palermo, Italy, from 01.01.2020 to 31.12.2022. For each of them, we recorded pertinent socio-demographic information, clinical data related to pregnancy, delivery and *peripartum*, and specifically execution and status of vaginal and rectal swab test for GBS detection, along with eventual administration and modality of IAP. The neonatal outcome was investigated in all cases at risk (positive maternal swabs status for GBS, either vaginal or rectal, with or without/incomplete IAP, preterm labor and/or delivery, premature rupture of membranes ≥ 18 h, previous pregnancy ended with neonatal early onset GBS disease [EOD], urine culture positive for GBS in any trimester of current gestation, *intrapartum* temperature ≥ 38 °C and/or any clinical/laboratory signs of suspected chorioamnionitis). The data concerning mothers and neonates at risk, observed during the pandemic (years 2020–2021), were compared with those of both subjects’ groups with overlapping risk factors recorded in the following period (year 2022). The chi squared test has been applied in order to find out the relationship between pregnant women with GBS colonization receiving IAP and outcome of their neonates.

**Results:**

The total source population of the study consisted of 2109 pregnant women, in addition to their 2144 newborns. Our analysis, however, focused on women and neonates with risk factors. The vaginal-rectal swab for GBS was performed in 1559 (73.92%) individuals. The test resulted positive in 178 cases overall (11.42% of those undergoing the screening). Amongst our whole sample of 2109 subjects, 298 women had an indication for IAP (vaginal and/or rectal GBS colonization, previous pregnancy ended with neonatal GBS EOD, urine culture positive for GBS in any trimester of current gestation, and unknown GBS status at labor onset with at least any among delivery at < 37 weeks’ gestation, amniotic membranes rupture ≥ 18 h and/or *intrapartum* temperature ≥ 38.0 °C), and 64 (21.48%) received adequate treatment; for 23 (7.72%) it was inadequate/incomplete, while 211 (70.8%) did not receive IAP despite maternal GBS colonization and/or the presence of any of the above mentioned risk factors. Comparing the frequency of performing vaginal-rectal swabs in the women admitted in the two time periods, the quote of those screened out of the total in the pandemic period (years 2020–2021) was higher than that of those undergoing GBS screening out of the total admitted in the year 2022 (75.65% vs. 70.38%, *p* = 0.009), while a greater number (not statistically significant, *p* = 0.12) of adequate and complete IAP was conducted in 2022, than in the previous biennium (26.36 vs. 18.62%). During the whole 3 years study period, as expected, none of the newborns of mothers with GBS colonization and/or risk factors receiving IAP developed EOD. Conversely, 13 neonates with EOD, out of 179 (7.3%) born to mothers with risk factors, were observed: 3 among these patients’ mothers performed incomplete IAP, while the other 10 did not receive IAP. Neither cases of neonatal meningitis, nor deaths were observed. The incidence rate in the full triennium under investigation, estimated as the ratio between the number of babies developing the disease out of the total of 2144 newborns, was 6.06‰; among those born to mothers with risk factors, if comparing the two time periods, the incidence was 8.06% in the pandemic biennium, while 5.45% in the following year, evidencing thus no statistical significance (*p* = 0.53).

**Conclusions:**

The present study revealed in our Department an increased prevalence of pregnant women screened for, and colonized by GBS, in the last decade. However, an overall still low frequency of vaginal-rectal swabs performed for GBS, and low number of adequate and complete IAP despite the presence of risk factors have been found, which did not notably change during the two time periods. Moreover, significant EOD incidence rates have been reported among children of mothers carrying risk factors, although also in this case no statistically significant differences have been observed during and after the pandemic. Such data seem to be in contrast to those reported during the COVID-19, showing a decrease in the access to health facilities and increased mortality/morbidity rates also due to the restrictive measures adopted to mitigate the effects of the pandemic. These findings might be explained by the presence within the same metropolitan area of our Department of a COVID hospital and birthing center, which all the patients with SARS-CoV-2 infection referred to, and likely leading to a weaker concern of getting sick perceived by our patients. Although IAP is an easy procedure to implement, however adherence and uniformity in the management protocols are still not optimal. Therefore, the prophylactic measures adopted to date cannot be considered fully satisfactory, and should be improved. Better skills integration and obstetrical-neonatological collaboration, in addition to new effective preventive tools, like vaccines able to prevent invasive disease, may allow further reduction in morbidity and mortality rates related to GBS perinatal infection.

**Supplementary Information:**

The online version contains supplementary material available at 10.1186/s13052-024-01738-2.

## Background

Group B *Streptococcus* (GBS), also known as *Streptococcus agalactiae* for its causative role in bovine mastitis, is present in the genitourinary and gastrointestinal tracts of pregnant women. Maternal GBS colonization rates vary worldwide from 10 to 40%, with mean prevalence of 18% [1–4]. In the newborn, GBS infection may give rise to both early (EOD) and late onset diseases (LOD). EOD is generally acquired by vertical transmission, and its most common clinical pictures include sepsis, pneumonia and meningitis. LOD, conversely, may be observed between 7 and 90 days of life, and the association with maternal colonization is not as strong as for EOD [5–7]. *Intrapartum* antibiotic prophylaxis (IAP), administrated ≥ 4 h before delivery, is the only currently available and highly effective method against early onset GBS neonatal infections. It allowed a reduction in the incidence of EOD of more than 80%, from 1.8 newborns per 1,000 live births in the 1990s to 0.23 newborns per 1,000 live births in 2015, although not being able to limit the impact of LOD [8, 9]. Since the onset of the coronavirus disease 2019 (COVID-19) pandemic, more than 6.9 million people died worldwide due to SARS-CoV-2 infection [10]. In Italy, it has been associated with significant clinical and psychological effects, also in pregnant women. Indeed, the infection control and the preventive measures (social distancing, mask wearing, hand hygiene and quarantine) implemented to mitigate the effects of the pandemic, led to the reduction in the access to many health facilities and services, including the obstetric and perinatal ones (only 28% of maternal and perinatal healthcare facilities continued to provide outpatient routine visits and examinations as usual, while 59% of them provided visits to a limited extent) [11]. Such decrease was linked both to the difficulties encountered by people in keeping the support from other family members within the hospital, and to the fear of contracting the infection [12, 13]. This additional negative impact of COVID-19 (besides the direct one caused by the infection) might have caused also worse health outcomes in pregnant women, especially in groups at major risk for social or economic reasons. The purpose of the present study was to retrospectively evaluate the prevalence rates of maternal vaginal-rectal GBS colonization, as well as use of IAP and incidence of episodes of neonatal GBS infection when antibiotic prophylaxis has not been carried out in colonized and/or at risk subjects, in a population of pregnant women during (years 2020–2021) and after (year 2022) the COVID-19 pandemic, also with the aim to establish possible epidemiological and clinical differences in the two subjects’ groups.

## Methods

We retrospectively analyzed the clinical data of pregnant women admitted to, and delivering, at the Gynaecology and Obstetrics Unit, Department of Sciences for Health Promotion and Mother and Child Care, of the University Hospital of Palermo, Italy, from 01.01.2020 to 31.12.2022. For each of them, we recorded the pertinent socio-demographic information, and the clinical data related to pregnancy, delivery and *peripartum*. Such items are detailed and presented in Table 1.

**Table 1:** Characteristics of the pregnant women analyzed

Variables
Age at delivery
Nationality
Residence
Occupation
Execution of vaginal and rectal swab test for GBS
Status of vaginal and rectal swab test for GBS
Type of delivery (i.e., vaginal, elective or emergency cesarean section)
Premature rupture of membranes (PROM, ≥ 18 h before delivery)
Eventual IAP

GBS: Group B *Streptococcus*; IAP: intrapartum antibiotic prophylaxis

We considered the results of vaginal and rectal swabs for GBS, performed between weeks 35^+ 0^ and 37^+ 0^ of gestation. This procedure is usually performed routinely in our Hospital in such time window, according to the indications of the Centers for Disease Control and Prevention (CDC) [8, 14]. Specifically, IAP was recommended in women with a positive GBS screening culture (excluding those undergoing cesarean delivery with intact amniotic membranes before labor onset), a previous child with early onset GBS disease, bacteriuria documenting GBS in the current pregnancy, and in those with unknown GBS status at labor onset and at least one of the following risk factors: delivery at < 37 weeks’ gestation, amniotic membranes rupture ≥ 18 h and/or *intrapartum* temperature ≥ 38.0 °C [15–17]. However, many variations of practice, based on the individual gynecologist and/or on mother’s compliance, have been observed during the study period in our sample population. Ampicillin was the first-line drug used, and it was administered intravenously (IV) at the dose of 2 g, followed by 1 g IV every 4 h until delivery. Cefazolin was the option chosen for women allergic to penicillin but not at high risk for anaphylaxis, while clindamycin or vancomycin have been used for high risk of anaphylaxis to penicillin, according to guidelines [18, 19]. IAP was considered adequate and complete when administered ≥ 4 h before delivery [8]. We focused on the rate of patients undergoing GBS screening, and on those with positive GBS screening tests. We also evaluated the indications for IAP, as well as the modality of IAP execution (if either adequate/complete or not).

The neonatal outcome was investigated in all cases at risk (positive maternal swabs status for GBS, either vaginal or rectal, with or without/incomplete IAP, preterm labor and/or delivery, premature rupture of membranes ≥ 18 h, previous pregnancy ended with neonatal EOD, urine culture positive for GBS in any trimester of current gestation, *intrapartum* temperature ≥ 38 °C, and/or any clinical/laboratory signs of suspected chorioamnionitis). In-depth data analyzed for each newborn are reported in Table 2.

**Table 2:** Clinical features of the newborns included in the analysis

Variables
Sex
Gestational age
Apgar scores at 1 and 5 min
Weight, length and occipitofrontal circumference at birth, and respective percentiles
Respiratory, metabolic and systemic disturbances; neurological manifestations and/or feeding problems
Blood cell count; C-reactive protein (CRP) and/or procalcitonin (PCT) values; blood culture results
Eventual admission to neonatal intensive care unit (NICU)
Eventual intravenous antibiotic therapy, its duration, and type of drugs used

The data observed during the pandemic (years 2020–2021) were compared with those recorded in the following period (year 2022).

### Statistical analysis

We used R version 4.0.4 (R Foundation for Statistical Computing, Vienna, Austria) for data analysis. Simple descriptive statistics were expressed as frequency and percentage for categorical variables, mean and standard deviation for continuous variables. Paired-samples t-test was used to compare data on the vaginal-rectal GBS colonization rate, as well as use of IAP and its effects on neonatal outcomes during (years 2020–2021) and after (year 2022) the COVID-19 pandemic. The Chi-squared test has been applied for comparison between two groups, and precisely in order to find out the relationship between pregnant women with GBS colonization receiving IAP and outcome of their neonates.

A *p* value lower than 0.05 was considered statistically significant.

## Results

### Socio-demographic information, and clinical data related to pregnancy, delivery and *peripartum*

The total number of deliveries observed during the study period was 2315, including 35 twin births (all bigeminal). The medical records were not available for 206 mothers, and therefore the source population of the study involved 2109 pregnant women, in addition to their 2144 newborns. Evaluated by year, the total number of delivering women was as follows: 660 in 2020, 757 in 2021, and 692 in 2022. There were 141 preterm (< 37 weeks of gestational age) deliveries, while the other 1968 were full-term ones. In the population under investigation, the average age was 30.42 ± 6 years, ranging between 15 and 52. Foreign mothers, defined as those who were not born in Italy, were the 12.94%. The most frequent countries of birth were Bangladesh (40.2%), Nigeria (15.01%), Morocco (7.69%), Romania (7.47%) and Tunisia (5.1%). The 69.41% of participants were resident in urban areas, while 30.59% came from rural ones. In regard with mothers’ occupation, 73.82% were housewives, 14.22% employees, 10.38% freelance professionals and 1.57% craftswomen/tradeswomen. In our sample, 1004 women (47.61%) had vaginal deliveries, while 650 (30.82%) and 455 (21.57%) underwent elective and emergency cesarean sections, respectively. 779 participants (36.94%) were primiparous. Sociodemographic and clinical data related to pregnancy, delivery and *peripartum* of the source population of women are summarized in Table 3.

**Table 3:** Sociodemographic and clinical data related to pregnancy, delivery and *peripartum* of the source population of women

Variables	*N* (%)
**Age (years)**	
Range	15–52
Mean ± SD	30.42 ± 6
**Nationality**	
Italian	1836 (87.06%)
Not Italian	273 (12.94%)
**Occupation**	
Housewives	1557 (73.82%)
Employees	300 (14.22%)
Freelance professionals	219 (10.38%)
Craftswomen/tradeswomen	33 (1.57%)
**Gestational age at delivery**	
< 37 weeks	141 (6.69%)
≥ 37 weeks	1968 (93.31%)
**Multiple pregnancy**	
Yes	35 (1.66%)
No	2074 (98.34%)
**Parity index**	
Primigravida	779 (36.94%)
Parity 1 or above	1330 (63.06%)
**Type of delivery**	
Vaginal	1004 (47.61%)
Elective cesarean section	650 (30.82%)
Emergency cesarean section	455 (21.57%)

SD: Standard deviations

### Prevalence rates of maternal GBS colonization and use of IAP

The vaginal-rectal swab for GBS was performed in 1559 (73.92%) individuals. More precisely, 512 were carried out in 2020, 560 in 2021 and 487 in 2022. The test resulted positive in 178 cases overall (11.42% of those undergoing the screening): 56 were those in 2020, 66 in 2021, and 56 in 2022.

Among GBS-positive patients, 41 (23.03%) received complete IAP, while to 20 (11.24%) an incomplete IAP was administered. 48 women (26.97%) did not receive IAP, due to cesarean sections performed before the onset of labor and with intact amniotic membranes; 69 subjects (38.76%), conversely, did not undergo IAP despite the presence of one or more clinical indications (Table 4).

**Table 4:** Frequency of vaginal-rectal swabs performed, prevalence rates of maternal GBS colonization, and use of IAP among GBS positive pregnant women

Variables	*N* (%)
**Execution of vaginal and rectal swab for GBS**	
Yes	1559 (73.92%)
No	550 (26.08%)
**Status of vaginal and rectal swab for GBS among patients undergoing the screening**	
Positive	178 (11.42%)
Negative	1381 (88.52%)
**IAP among GBS positive patients**	
Complete	41 (23.03%)
Incomplete	20 (11.24%)
No IAP	117 (65.73%)
• due to cesarean sections performed before the onset of labor and with intact amniotic membranes	48 (26.97%)
• despite the presence of one or more clinical indications	69 (38.76%)

GBS: Group B *Streptococcus*; IAP: intrapartum antibiotic prophylaxis

Of the 550 (26.08%) pregnant women with unknown GBS colonization status, 120 (21.82%) had *intrapartum* risk factors. In this group, preterm delivery (< 37 weeks of gestation) was the only risk condition in 65 patients (11.82%), PROM ≥ 18 h in 43 (7.82%), while 12 (2.18%) of them had both risk factors (preterm delivery and PROM ≥ 18 h). No women presented with fever and/or other signs of chorioamnionitis. Considering only the individuals with *intrapartum* risk factors, 23 (19.17%) received complete IAP, the prophylaxis was incomplete in 3 (2.5%) cases, and for 94 (78.33%) it was not administered (Table 5).

**Table 5:** *Intrapartum* risk factors among pregnant women with unknown GBS colonization status and use of IAP

Variables	*N* (%)
***Intrapartum*** **risk factors** among pregnant women with unknown GBS status	
Yes	120 (21.82%)
No	430 (78.18%)
**Type of** ***intrapartum*** **risk factors**	
Preterm delivery	65 (11.82%)
PROM ≥ 18 h	43 (7.82%)
Both preterm delivery and PROM ≥ 18 h	12 (2.18%)
Fever and/or other signs of chorioamnionitis	0 (0%)
**IAP among GBS pregnant women with unknown GBS status and** ***intrapartum*** **risk factors**	
Complete	23 (19.17%)
Incomplete	3 (2.5%)
No IAP	94 (78.33%)

GBS: Group B *Streptococcus*; IAP: intrapartum antibiotic prophylaxis

Amongst our overall sample of 2109 subjects, 298 women had an indication for IAP (vaginal and/or rectal GBS colonization, previous child with EOD, bacteriuria documenting GBS in the current pregnancy, and unknown GBS status at labor onset and at least any among delivery at < 37 weeks’ gestation, amniotic membranes rupture ≥ 18 h and/or *intrapartum* temperature ≥ 38.0 °C), and 64 (21.48%) received adequate treatment; for 23 (7.72%) it was inadequate/incomplete, while 211 (70.8%) did not receive IAP despite maternal GBS colonization and/or the presence of any of the above mentioned risk factors. Most cases where the prophylaxis was indicated, but in which it was not performed or was inadequate/incomplete, were represented by pregnant women admitted to hospital in advanced labor or presenting with precipitous delivery. In a few subjects IAP was simply omitted, probably for misinterpreted/incorrect data on GBS swabs at the time of birth.

Comparing the Italian mothers with the foreign ones, a higher (*p* < 0.0001*)* frequency of vaginal-rectal swabs for GBS was found in the whole period under investigation among the former (75.49% vs. 63.37%), as well as a greater number (although not statistically significant, *p* = 0.72) of adequate and complete IAP (21.86% vs. 19.61%). Conversely, the rate of positive GBS swabs was significantly higher among the foreign mothers (10.46% in the group of Italian women vs. 19.08% in the latter, *p* = 0.0008). Comparing the frequency of performing vaginal-rectal swabs in the women admitted in the two time periods, the quote of those screened out of the total in the pandemic period (years 2020–2021) was higher than that of those undergoing GBS screening out of the total admitted in the year 2022 (75.65% vs. 70.38%, *p* = 0.009), while a greater number (however not statistically significant, *p* = 0.12) of adequate and complete IAP was conducted in 2022, than in the previous biennium (26.36 vs. 18.62%). Finally, the comparison between the periods during and after COVID-19 revealed a mildly lower (without statistical significance, *p* = 0.94) GBS colonization rate during the pandemic than the following year (11.38% vs. 11.5%; Table 6).

**Table 6:** Comparison of data observed during (years 2020–2021) and after (year 2022) the COVID-19 pandemic

Items	During the pandemic (2020–2021) (%)	After the pandemic (2022) (%)	*P* value
Execution of vaginal-rectal swabs	75.65%	70.38%	<0.009*
Adequate and complete IAP	18.62%	26.36%	0.12
GBS colonization rate	11.38%	11.5%	0.94

GBS: Group B *Streptococcus*; IAP: intrapartum antibiotic prophylaxis

* Statistically significant difference between during and after the COVID-19 pandemic

### Effects of maternal GBS colonization in the newborn

During the study period, as expected, none of the newborns of mothers with GBS colonization and/or risk factors receiving IAP developed EOD. Conversely, 13 neonates with EOD, out of 179 (7.3%) born to mothers with risk factors (including overall those showing positive, negative, and unknown GBS status, i.e. 60, 11 and 108 respectively), were observed: the incidence rate, estimated as the ratio between the number of babies developing the disease out of the total of 2144 newborns delivered in the 3 years studied, was 6.06‰. Neonatal sepsis was noted in 10 babies born to 121 mothers who did not perform IAP (8.2%), and in 3 neonates born to 19 women whose prophylaxis was incomplete (15.7%). Furthermore, EOD incidence in the COVID-19 period was 8.06% (10 cases out of 124 women with risk factors), while that of the post-pandemic year analyzed was 5.45% (3/55 born to mothers at risk), without a statistically significant difference between the two time periods (*p* = 0.53). Among the infected neonates, 9 were male and 4 female. Mean gestational age was 39^+ 4^ weeks. All newborns had normal Apgar scores (> 7) at 1 and 5 min. The average birth weight was 3249 ± 482 g, length 49.6 ± 2.7 cm, and occipitofrontal circumference 34.0 ± 1.5 cm. 2 of them were small for gestational age (SGA), while 11 were appropriate for gestational age (AGA).

**Fig. 1:**
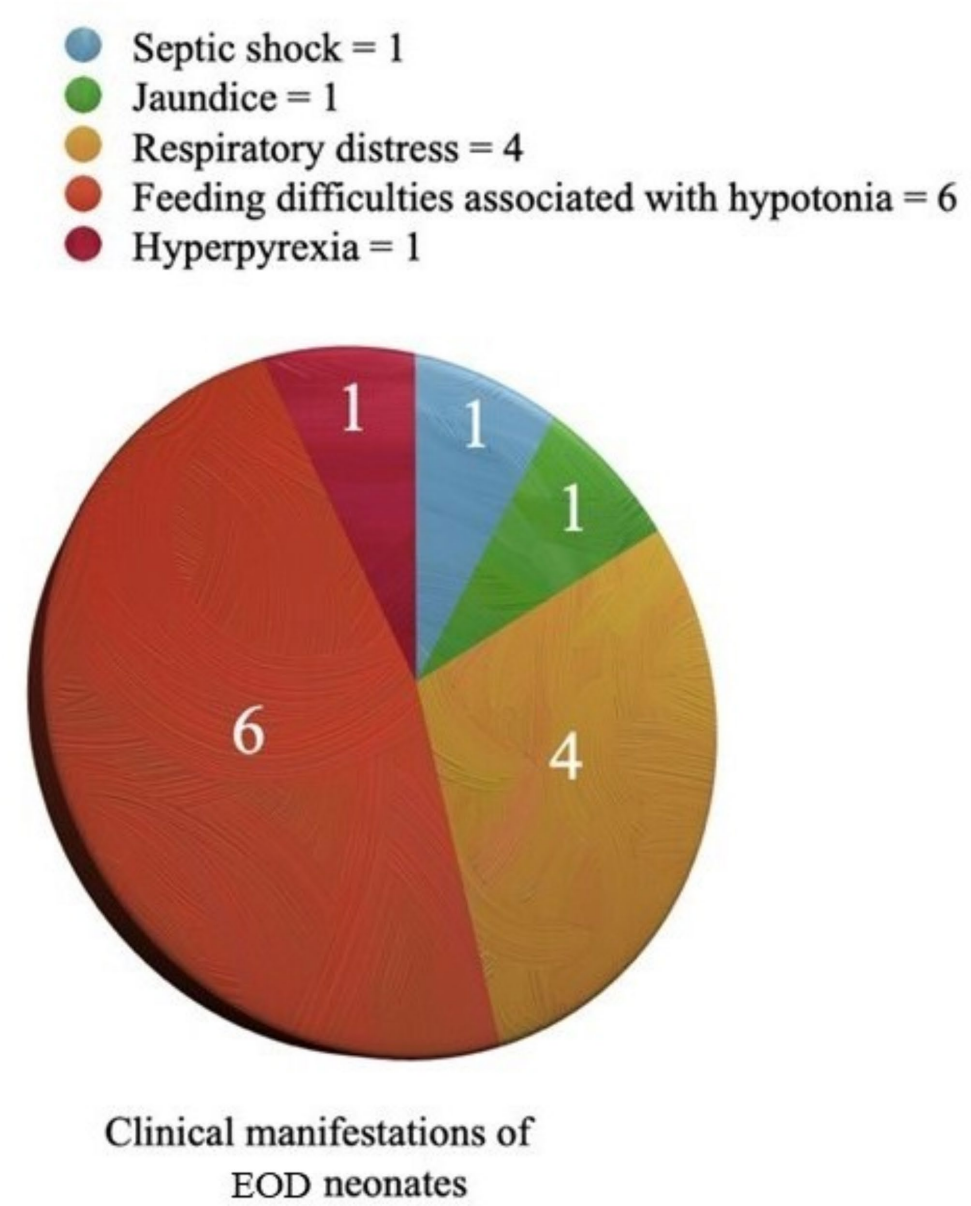
Clinical manifestations of EOD neonates

Clinical manifestations included septic shock (1), jaundice (1), respiratory distress (4), feeding difficulties/regurgitation associated with hypotonia (6), while hyperpyrexia was present in 1 case (Fig. 1).

Increased inflammation indices (CRP and/or PCT) were detected in all newborns. Blood cultures were carried out in all subjects before the start of antibiotic therapy, and resulted negative in all cases. 4 subjects required hospitalization in the NICU, while in 9 cases the admission to the Neonatal Pathology Unit (sub-intensive care setting) was necessary. The patients were hospitalized for an average of 11 ± 3 days. The mean duration of antibiotic therapy was 7 ± 3 days. Empiric therapy with ampicillin (100 mg/kg/dose every 12 h) and gentamicin (4 mg/kg/dose every 24 h) was promptly started in all neonates. The antimicrobial treatment was continued until clinical symptoms disappeared, as well as complete blood counts, inflammation indices, and blood culture tests gave normal/negative results. There was no evidence of meningitis in any case, and no deaths were observed (Table 7).

**Table 7:** Clinical data of EOD neonates

Variables	*N* (%)
**Sex**	
Male	9 (69.23%)
Female	4 (30.77%)
**Gestational age at birth (weeks)**	
Mean	39^+ 4^
**Apgar score at 1 min**	
< 7	0 (0%)
≥ 7	13 (100%)
**Apgar score at 5 min**	
< 7	0 (0%)
≥ 7	13 (100%)
**Birth weight (g)**	
Mean ± SD	3249 ± 482
**Length at birth (cm)**	
Mean ± SD	49.6 ± 2.7
**Occipitofrontal circumference at birth (cm)**	
Mean ± SD	34.0 ± 1.5
**Small for gestational age**	
Yes	2 (15.38%)
No	11 (84.62%)
**IAP**	
Complete	0 (0%)
Incomplete	3 (23.08%)
No IAP	10 (76.92%)
**Hospitalization**	
NICU	4 (30.77%)
Neonatal Pathology Unit (sub-intensive care setting)	9 (69.23%)
**Length of hospital stay (days)**	
Mean ± SD	11 ± 3
**Treatment**	
Ampicillin + Gentamicin	13 (100%)
**Duration of antibiotic therapy (days)**	
Mean ± SD	7 ± 3

IAP: intrapartum antibiotic prophylaxis; SD: standard deviations; NICU: Neonatal intensive care unit

## Discussion

**Fig. 2a/b:**
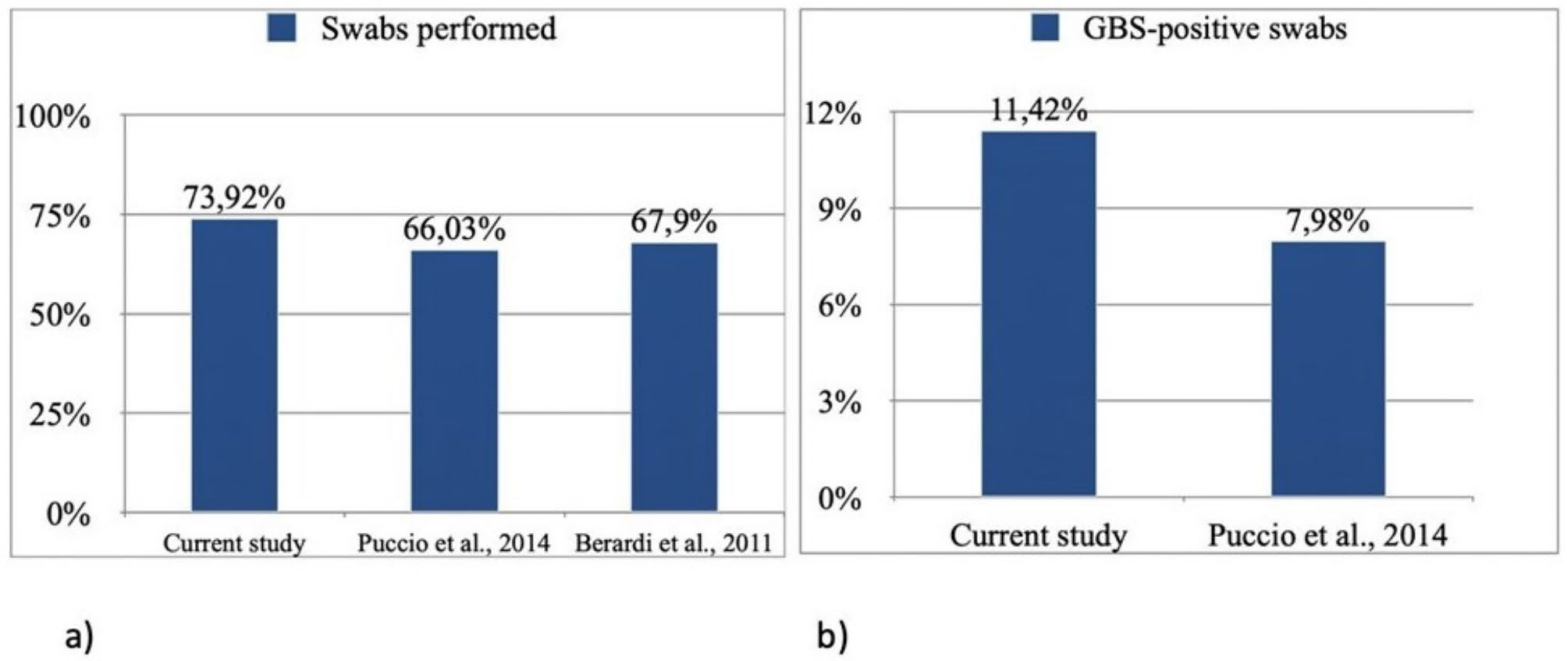
Comparison of GBS screening among the current study and those previously reported in our Hospital and in Central Italy **(a)**, and of maternal GBS colonization between the present analysis and that conducted by Puccio et al. in 2012 in our Department **(b)**

Group B Streptococcus is a major cause of invasive infections in neonates, with the colonization of the vaginal-rectal tract of pregnant women being the main transmission source. Our data provide updated insights about the prevalence of vaginal-rectal GBS colonization in pregnancy. In addition, the present study shows the rates of adhesion to GBS screening and to IAP in a cohort of pregnant women referring to a II level University Hospital in the city of Palermo, Italy. In our sample, the quote of subjects screened for GBS (in all of them a complete vaginal–rectal swab was performed) out of the total addressed to our Mother and Child Department was 73.92%. Such data were higher than those of a previous retrospective study carried out in our Hospital in 2012, and also than the rates recorded by Berardi A. et al. in 2011 in Central Italy, which were 66.03% and 67.9% respectively (Fig. 2a) [20, 21]. According with CDC and the Italian Obstetrics Society guidelines, the execution of vaginal–rectal cultures for GBS is recommended between 35 and 37 weeks of gestation, and such indications were those followed also in the present study [8, 14]. In our population vaginal and rectal swabs were positive for GBS in the 10.42% of cases; this value is at the lower range of the national average, which is between 10 and 20% [22]. Comparing the current analysis with that carried out in 2012 in our Hospital [20], an increased prevalence of GBS colonization in our population has been observed in the last few years (from 7.98 to 11.42%) (Fig. 2b) [19].

Worldwide, frequencies of maternal GBS carriers have been reported to range from 14 to 30% in high-income countries (mildly higher than the present survey), to be around 19% in the Sub-Saharan region, and 12–15% in India and Pakistan [23–25]. Differences in the detected rate of vaginal-rectal GBS colonization may reflect the different demographic characteristics of the populations under investigation. Actually, GBS incidence rates can vary, either according to geographical region or time period [26]. Indeed, when comparing COVID-19 with the post-pandemic *scenario*, we detected a mild decrease in GBS maternal colonization during the years 2020–2021 (11.38% vs. 11.5%).

Amongst our overall sample, only 21.48% women received adequate IAP in presence of clinical indications (positive GBS screening culture or *intrapartum* risk factors). The consequent higher rate of subjects who did not receive or performed incomplete/inadequate IAP can be due to those women admitted in advanced labor or presenting with a precipitous one, in addition to the few cases in which it was omitted for misinterpreted/incorrect data on GBS status at delivery. Such gap is a critical issue which clinicians must be focused on, aiming at reducing the preventable maternal and neonatal adverse outcomes, implementing awareness, antenatal care programs and dedicated operative Department protocols. Indeed, in Central Italy a major proportion (> 90%) of individuals showing GBS-positive cultures received adequate treatment [21]. In the USA, the prevalence of mothers with an indication for IAP who received adequate treatment increased, from 73.8% between 1998 and 1999 to 85.1% between 2003 and 2004 [27, 28]. Comparing the pandemic period (years 2020–2021) with the following one (2022), a higher frequency in the execution of vaginal-rectal swabs for GBS and a lower (although not statistically significant) of adequate and complete IAP, were found in the first two years than in 2022. Actually, despite the infection control and preventive measures adopted to lessen the pandemic’s effects resulted in a decrease in the access to various health facilities, including obstetric and perinatal care services, however in our care setting such reduction was not observed, probably due to the presence within the same metropolitan area of our Department, of a dedicated COVID hospital and birthing center (as documented also by the decrease of the total number of deliveries evidenced during the year 2022, corresponding to the suspension of the COVID hospital activity and its reconversion to regular health care), which all patients with SARS-CoV-2 infection referred to. Therefore, it may be likely that the reduction in the health care accesses reported during the pandemic did not occur in the present experience due to a weaker concern of getting sick perceived by our patients, as well as to the availability offered in our facility to maintain the support of other family members during hospital stay and antenatal care visits [11–13]. Finally, we detected inequalities between the Italian women and the foreign ones due to the major number of swabs performed among the former and, although not statistically significant, higher colonization rates in the latter.

We reported an EOD incidence of 7.69% among children of mothers carrying risk factors, and of 6.06‰ out of the total number of newborns delivered during the 3-year investigation (i.e., *n* = 2144). In our study the clinical picture of the early form of disease was represented by sepsis. According to literature, respiratory signs were the initial most common typical symptoms, only preceded by poor feeding/regurgitation associated with hypotonia, frequently described in literature reports as well [29]. The other less common clinical manifestations identified were fever, jaundice, and septic shock, which are not typical of GBS, and which can occur in other bacterial infections. Mortality is estimated to be 2–5% in full-term children, and increases by 25% in preterm infants; nonetheless, in our sample (in which, however, no preterm babies were present) neither deaths nor meningitis were documented [30, 31]. It is noteworthy that, as expected, none of the mothers’ patients received adequate/complete IAP, with evidence of EOD both in the group of those born to women with non-performed (8.2%) or incomplete (15.7%) prophylaxis. These data further highlight how relevant could be to begin IAP as soon as possible, when a clinical indication is identified, due to the beneficial effects of a prompt IAP (at least four hours before birth).

Our results demonstrate that there is still a relevant number of women who do not perform appropriate IAP despite being properly identified as colonized with GBS at delivery, in addition to those who are not even recognized as GBS-positive by antenatal screening cultures. The identification and treatment of candidates for IAP are necessary, as moreover evidenced by the present study, also owing to the higher risk of developing EOD for neonates born to mothers without GBS screening and not receiving adequate and/or complete IAP. In order to stop and/or limit GBS infections, local public health organizations should support both microbiological surveillance and educational initiatives [32, 33]. These interventions, actually, are able to reduce by 80% the risk of neonatal sepsis or meningitis, specifically early onset ones, i.e. those between birth and the completion of the 6th day of life [34–36]. Indeed, such strategies cannot be effective in the remaining 20% of early infections, as they are not linked to fetal contamination with the bacteria encountered during the passage through the vaginal canal at birth. They are, rather, dependent on infections contracted prior to the delivery, due to the ascending passage of germs to the fetus, especially in case of premature rupture of membranes. Although the total number of cases of neonatal GBS infection is not reported to be overly high, as highlighted also in the present analysis, however it is clear that the prophylaxis measures adopted to date cannot be considered fully satisfactory. Pregnant woman screening, indeed, is not always easy to implement, as well as the administration of *intrapartum* antibiotics, which often does not follow in the clinical daily practice (as evidenced in our experience), the effective modalities established by CDC guidelines for the eradication of the bacterium. Clinicians, then, need to be careful and accurate in the correct adhesion to care protocols, also in consideration of the high number of inadequate and/or missing IAP administrations, as documented by the present analysis. In addition to the implementation and improvement of antibiotic prophylaxis, however, the search for alternative preventive tools, such as the production of an effective and safe vaccine administered to the mother, appears urgent and not postponable [37–40].

## Conclusions

The present study revealed in our Department an increased prevalence of pregnant women screened for, and colonized by GBS, in the last decade. However, an overall still low frequency of vaginal-rectal swabs performed for GBS, and low number of adequate and complete IAP despite the presence of risk factors have been found, which did not notably change during the two time periods. Moreover, relevant EOD incidence rates have been reported among children of mothers carrying risk factors, although no statistically significant differences have been observed during and after the COVID-19. Such data seems to be in contrast with those observed during the pandemic for other care settings (especially emergency care areas, as well as surgery and diagnostic services), where notable delays in diagnosis and treatment, and increase in mortality/morbidity rates due to the indirect effects of COVID-19 (reduction in the number of clinical checks, fear in the access to health facilities) have been described. However, in our care setting such findings were not observed, probably due to the presence, within the same metropolitan area of our Department, of a dedicated COVID hospital and birthing center, which all subjects with SARS-CoV-2 infection referred to. This likely led to a weaker concern of getting sick perceived by our patients, as well as to the availability offered in our facility to maintain the support of other family members during the hospital stay and the antenatal care visits. Furthermore, inequalities in the number of swabs performed persist, compared to the past, between Italian and foreign women, highlighting an insufficient health support provided to migrant and at risk populations [32].

Although IAP is an easy procedure to implement, and our population of women subjected to screening increased in the last years, nonetheless adherence and uniformity of its management protocols are still not optimal. Despite the total number of neonatal GBS infections is not reported to be overly high, as documented also in the present analysis, however the prophylactic measures adopted to date cannot be considered fully satisfactory, and therefore should be improved. Better skills integration and obstetric-neonatological collaboration, in addition to new effective preventive tools, like vaccines [41, 42] able to prevent invasive disease, may allow further reduction in morbidity and mortality rates related to GBS perinatal infection.

## Electronic supplementary material

Below is the link to the electronic supplementary material.

Supplementary Material 1

## Data Availability

The datasets used and analyzed during the current study are available from the corresponding author on reasonable request.

## Abbreviations

CDC: Centers for Disease Control and Prevention
COVID-19: Coronavirus disease 2019
CRP: C-reactive protein
EOD: Early onset disease
GBS: Group B Streptococcus
IAP: Intrapartum antibiotic prophylaxis
IV: Intravenous
LOD: Late onset disease
NICU: Neonatal intensive care unit
PCT: Procalcitonin
PROM: Premature rupture of membranes
