# Supplementary material for: Group B streptococcus colonization in pregnancy and neonatal outcomes: a three-year monocentric retrospective study during and after the COVID-19 pandemic
Source: Ital J Pediatr. 2024 Sep 13;50:175. doi: 10.1186/s13052-024-01738-2 (PMC11395677; doi:10.1186/s13052-024-01738-2)
Supplement: Supplementary file 1 — Supplementary Material 1 [file 13052_2024_1738_MOESM1_ESM.docx]

N

Gregorio Serra, M.D.

Department of Health Promotion, Mother and Child Care,

Internal Medicine and Medical Specialties “G. D’Alessandro”

University of Palermo

Piazza delle Cliniche, 2

90127 Palermo, Italy

Dear Editor,

# My colleagues and I are submitting a manuscript entitled “Group B streptococcus colonization in pregnancy and neonatal outcomes: a three-year monocentric retrospective study during and after the COVID-19 pandemic”, for consideration by Italian Journal of Pediatrics.

We confirm that this work is original and has not been published elsewhere, nor it is currently under evaluation for publication elsewhere.

The purpose of the present study was to retrospectively evaluate the prevalence rates of maternal vaginal-rectal Group B *Streptococcus* colonization, as well as use of *intrapartum* antibiotic prophylaxis (IAP) and incidence of episodes of neonatal GBS infection when antibiotic prophylaxis has not been carried out in colonized and/or at risk subjects, in a population of pregnant women during (years 2020-2021) and after (year 2022) the COVID-19 pandemic.

The present study revealed in our Department an overall still low frequency of vaginal-rectal swabs performed for GBS, and low number of adequate and complete IAP despite the presence of risk factors, which did not notably change during the two time periods. Moreover, significant EOD incidence rates have been reported among children of mothers carrying risk factors, with no statistically significant differences observed during and after the pandemic. Although IAP is an easy procedure to implement, however adherence and uniformity in the management protocols are still not optimal. Therefore, the prophylactic measures adopted to date cannot be considered fully satisfactory, and should be improved.

The paper should be of interest to readers in the areas of Pediatrics and Infectious Diseases. Please address all correspondence about the manuscript to me, as corresponding author, at gregorio.serra@unipa.it

Thank you for your consideration of this manuscript.

Sincerely,

Gregorio Serra

Palermo, 15/07/2024
